# Complete response of metastatic cutaneous squamous cell carcinoma and multiple locally advanced basal cell carcinomas with concomitant pembrolizumab and sonidegib therapy

**DOI:** 10.1016/j.jdcr.2024.02.011

**Published:** 2024-03-01

**Authors:** Carlos González-Cruz, Eva Muñoz-Couselo, Carolina Ortiz-Velez, Berta Ferrer, Vicente García-Patos, Carla Ferrándiz-Pulido

**Affiliations:** a Department of Dermatology, Hospital Universitari Vall d’Hebron, Barcelona, Spain; b Facultad de Medicina, Universitat Autònoma de Barcelona, Barcelona, Spain; c Department of Medical Oncology, Vall d’Hebron Institute of Oncology, Hospital Universitari Vall d’Hebron, Barcelona Spain; d Department of Pathology, Hospital Universitari Vall d’Hebron, Barcelona, Spain

**Keywords:** basal cell carcinoma, hedgehog, immunomodulatory therapy, squamous cell carcinoma

## Introduction

The treatment of patients with metastatic cutaneous squamous cell carcinoma (cSCC) who also have multiple locally advanced (la) basal cell carcinomas (BCCs) can be challenging and requires multidisciplinary management. We present our clinical experience with concomitant treatment with pembrolizumab and sonidegib in a patient with metastatic cSCC and multiple locally advanced BCCs.

## Case report

**Fig 1:**
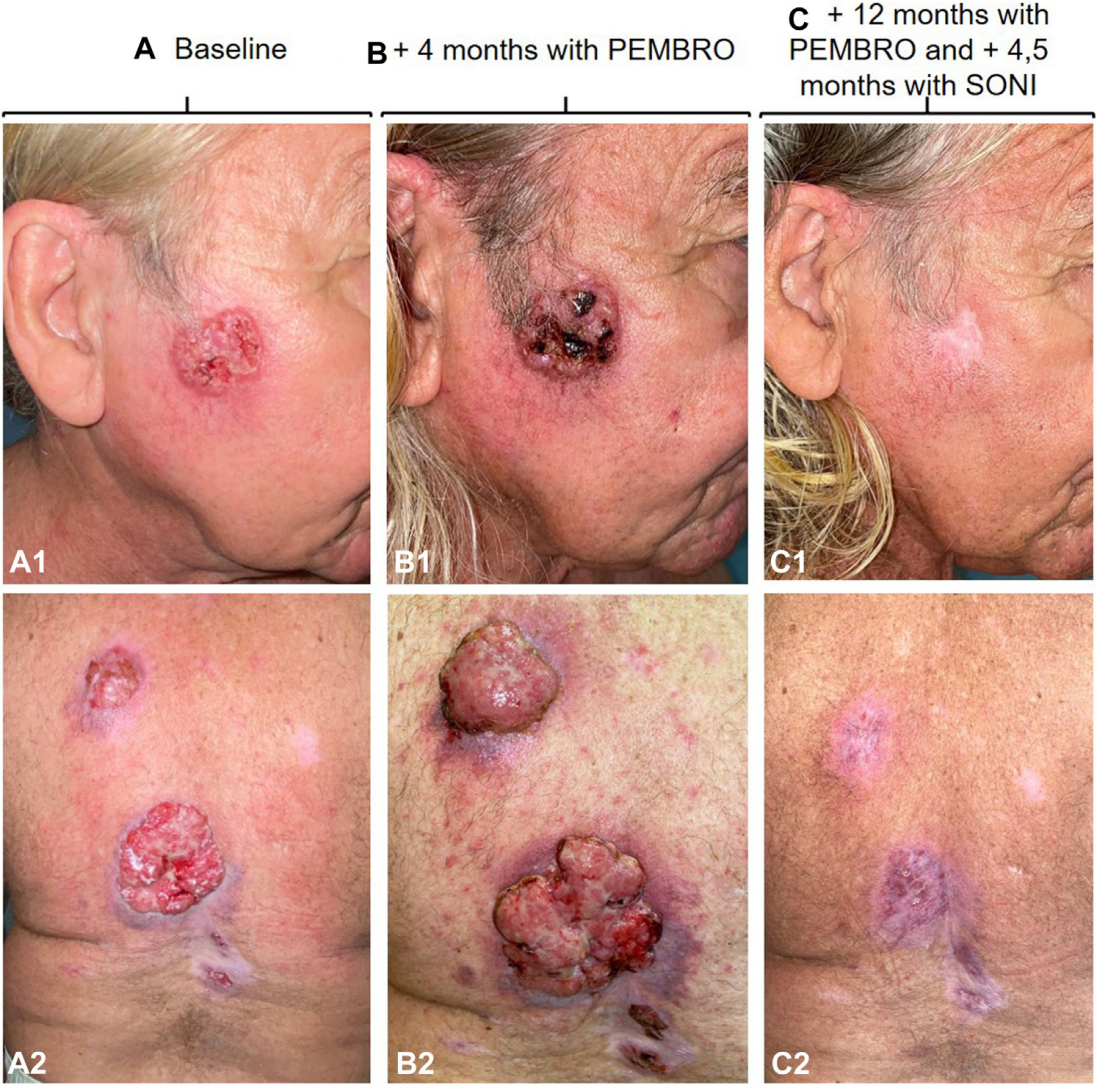
Locally advanced basal cell carcinoma (BCC) on (**A1**) cheek and (**A2**) back. **B,** BCCs pseudoprogression after 4 months of pembrolizumab. **C,** BCCs complete response after 4.5 months with sonidegib treatment and 12 months with pembrolizumab.

**Fig 2:**
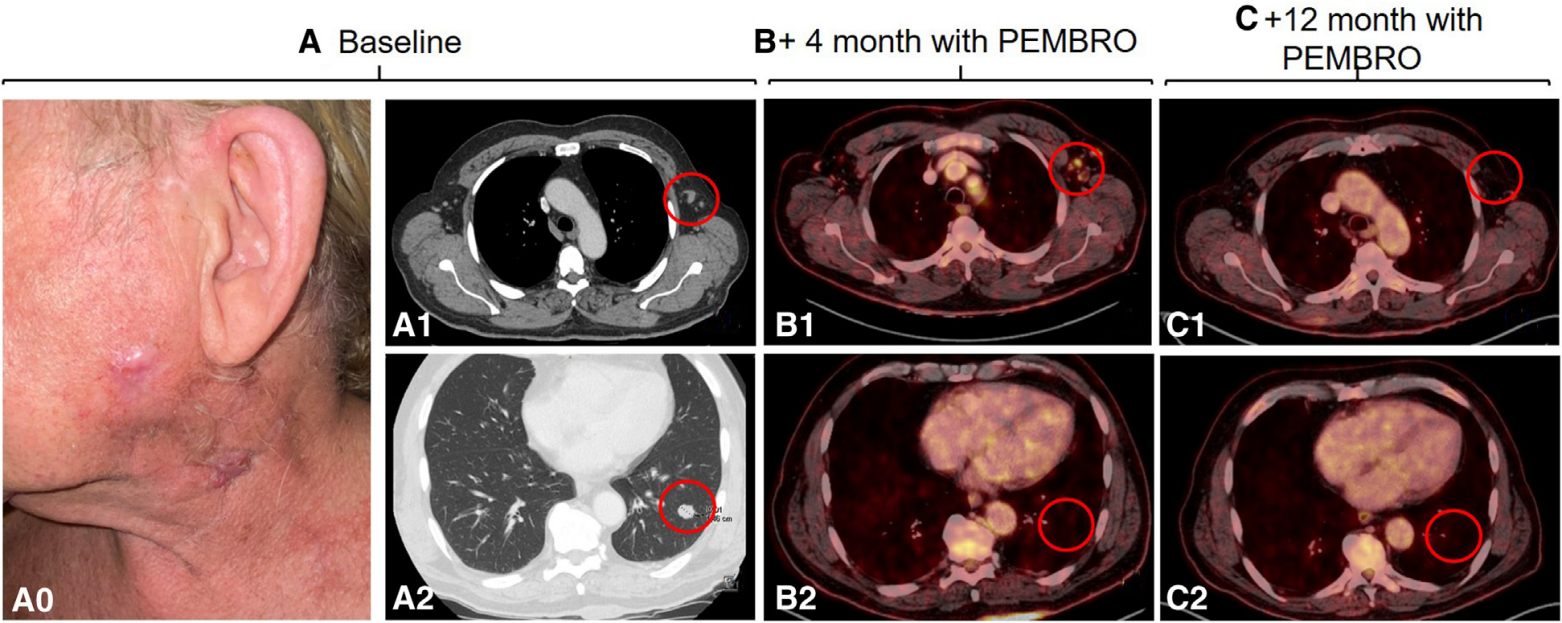
**A**, Baseline left cervical cutaneous squamous cell carcinoma. **A0,** Clinical image; **A1,** axillary lymph nodes by CT; **A2,** pulmonary metastases by CT. **B,** Dissociate response after 4 months of pembrolizumab treatment. **B1,** Lymph node pseudoprogression in PET/CT; **B2,** complete response of the lung metastases by PET/CT. **C,** Complete response after 12 months with pembrolizumab treatment. **C1,** Complete response of axillary lymph nodes by PET/CT; **C2,** complete maintained response of the lung metastases by PET/CT. *PET*, Positron emission tomography; *CT*, computed tomography.

A 73-year-old man with a history of occupational exposure to mica, asbestos, rock fiber, nickel, and chromium, with multiple BCCs previously treated since the age of 40 years, was referred to our center for evaluation of keratinocyte carcinomas. Following our assessment, 18 BCCs were diagnosed located mainly on the head and trunk (Fig 1, *A*). Three of them were locally advanced with a diameter of >5 cm, a vegetate growth, ulcerated and invading deep structures. In addition, the patient was diagnosed with a left cervical poorly differentiated cSCC, which was completely excised for histologic diagnosis. A computed tomography scan confirmed the presence of cervical and axillary lymph nodes and pulmonary metastases, which proved to have the same histology than the cSCC (Fig 2, *A*) (T3N3M1, American Joint Committee on Cancer eighth edition).

The interdisciplinary tumor board decided to initiate pembrolizumab at a dose of 200 mg every 21 days for the treatment of both, the metastatic cSCC and the multiple BCCs. The patient presented an initial dissociated response, with regression of the lung metastases after 4 months of treatment but with lymph node and BCCs initial pseudoprogression (Figs 1, *B* and 2, *B*). After 8 months of treatment, the patient achieved almost radiologic complete response of the cSCC metastases, with an excellent tolerance to the treatment and only arthralgia grade 1. The response of BCCs was, however, very modest. The clinical decision was to add sonidegib 200 mg daily for the treatment of the BCCs. The patient presented a clinical complete response after 4.5 months of combined treatment (Fig 1, *C*), when both treatments were discontinued, due to transient elevation of grade 2 transaminases. As the complete response of the BCCs was confirmed by histologic examination, sonidegib was not reinitiated. Treatment with pembrolizumab was reinitiated and discontinued after 15 months since its initiation, presenting radiologic complete response maintained in both squamous cell carcinoma (SCC) and BCC (Figs 1, *C* and 2, *C*). The reason for discontinuation was a transient elevation of troponins without clinical manifestations (requested in a routine analysis) which was attributed to immunotherapy. At the present time, 24 months after the start of pembrolizumab and 8 months after cessation of both treatments, the patient maintains a complete response of both tumors.

## Discussion

In recent years, the treatment of advanced and metastatic cSCC and laBCC has undergone a significant improvement.^1^^,^^2^

Limited case reports describe patients with both, advanced SCC and advanced BCCs exhibiting improved responses when treated sequentially with cemiplimab and sonidegib.^3^^,^^4^ The authors consider that the combination of both treatments is well tolerated, being the sonidegib-related toxicities grade 1 and 2.^4^

Our patient presented an even greater therapeutic challenge. He had a metastatic cSCC and multiple laBCCs. Furthermore, cemiplimab treatment is not funded in our country for the treatment of nonmelanoma skin cancer. Based on efficacy studies of pembrolizumab in the treatment of advanced cSCC and BCC,^5^^,^^6^ the interdisciplinary tumor board decided to initiate this treatment on a compassionate use basis, which achieved a complete response in metastatic cSCC. However, the response of BCC was not significant, and sonidegib was added to the treatment, and the patient achieved a complete response after only 4.5 months of treatment. As described in the aforementioned reports, we found that this combination was well tolerated and had a high synergistic effect.

To our knowledge, this is the first clinical report on the concomitant and sequential administration of pembrolizumab and sonidegib in a patient with metastatic cSCC synchronous to multiple locally advanced BCC, achieving complete response of all tumors with good tolerance to the combined treatment. BCC and cSCC are 2 different cancers and in our case immunotherapy demonstrated less efficacy in BCC than in cSCC. Immunotherapy is only indicated in laBCC in second line after progression to hedgehog inhibitors or after unacceptable toxicities to them.^7^

## Conflicts of interest

Dr Ferrándiz-Pulido has participated in advisory boards and/or received honoraria from Sun Pharma and Sanofi. Dr Muñoz-Couselo has participated in advisory boards for Amgen, Bristol-Myers Squibb, Merck Sharp & Dohme, Novartis, Pierre Fabre, Roche, and Sanofi, received honoraria from Amgen, Bristol-Myers Squibb, Merck Sharp & Dohme, Novartis, Pierre Fabre, and Roche, and participated as principal investigator in clinical trials for Amgen, Bristol-Myers Squibb, Merck Sharp & Dohme, Novartis, Pierre Fabre, Roche, and Sanofi. The other authors have no conflicts of interest to declare.
